# Dioxins, PFOS, and 20 other Persistent Organic Pollutants in Eggs of Nine Wild Bird Species from the Vaal River, South Africa

**DOI:** 10.1007/s00244-024-01088-4

**Published:** 2024-09-19

**Authors:** Velesia Lesch, Rialet Pieters, Hindrik Bouwman

**Affiliations:** Research Unit: Environmental Sciences and Management, North-West University, Potchefstroom, South Africa

**Keywords:** PCB, PBDE, PCDD/F, DDT, Pesticide, Heron, Ardeidae

## Abstract

The Vaal River catchment drains the largest and most populated industrial and mining region in Southern Africa. Heron, ibis, cormorant, egrets, and darter eggs, representing three habitats and four feeding guilds, were collected at four locations in 2009/10 to identify hotspots and hazards associated with persistent organic pollutants (POPs). The POPs included 21 organochlorine pesticides, five polybrominated diphenyl ether (PBDE) classes, 18 polychlorinated biphenyls (PCBs including six non-dioxin-like PCBs; NDL-PCB), and 12 dioxin-like PCBs (DL-PCBs), 17 polychlorinated dibenzo-p-dioxins and dibenzo-p-furans (PCDD/Fs), and perfluorooctane sulfonate (PFOS). Aquatic predators had higher PFOS and PCDD/F concentrations, while PCBs dominated in terrestrial eggs. Organochlorine pesticides, PBDEs, and PCBs were strongly associated with eggs from the industrial regions, while PCDD/F concentrations were evenly distributed. PCDD/F and PCB toxic equivalency quotient concentrations were low with no adverse effects expected. PFOS peaked at Bloemhof Dam with a maximum of 2300 ng/g wm in an African Darter egg, indicating an unexpected PFOS hotspot, the source of which is unknown. Despite order of differences in compound class concentrations, there was no association with egg size. To the best of our knowledge, this is the only study that analysed all 2010 POPs in bird eggs on a large geographic scale. This study highlighted the importance of multi-species studies sampling from multiple locations to assess the risk that POPs pose to avian populations as hotspots and species at risk may be missed by studies looking at one or few species.

**Supplementary Information:**

The online version contains supplementary material available at 10.1007/s00244-024-01088-4.

## Introduction

**Fig. 1:**
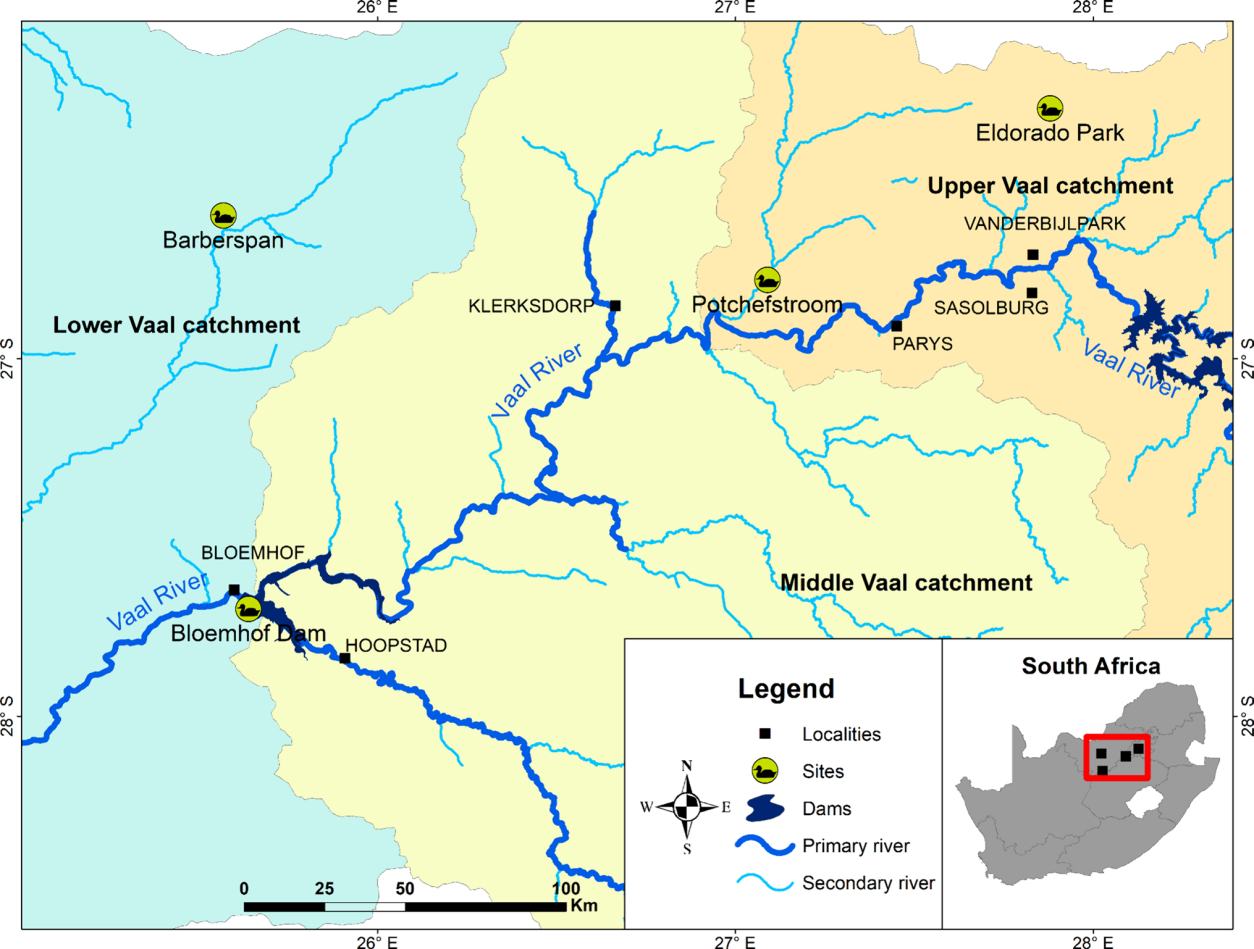
Map showing the wild bird egg sampling locations

One of the Southern Africa’s largest rivers, the Vaal River, flows westwards from Mpumalanga province to the Atlantic (Fig. 1). It flows through South Africa’s most industrialised regions before passing through rural and agricultural areas. The Vaal River merges with the Orange-Senqu River near the town of Douglas, forming the Orange-Senqu River Basin (OSRB), that stretches over four countries (Botswana, Lesotho, Namibia, and South Africa) covering approximately 1 000 000 km^2^ (Lange et al. 2007). The Orange River mouth at the South Atlantic Ocean was once a flourishing wetland with over 20 000 resident water birds and attracting many migrant birds. However, the number of resident birds has drastically decreased (Anderson et al. 2003).

There are seven Ramsar sites located in the OSRB (Orange River Mouth, Lets'eng-la-Letsie, Barbers Pan, Blesbok Spruit, Kgaswane Mountain Reserve, Seekoeivlei Nature Reserve, and Ingula Nature Reserve; Ramsar Sites Information Service. 2018b). Southern Africa is, however, a water-scarce region; many rural households, agriculture, mining, and industry directly make use of the OSRB’s surface and groundwater. The influx of agricultural and industrial products (including persistent organic pollutants, POPs) is a major cause of concern (Chokwe et al. 2019; Groffen et al. 2021; Gilbert et al. 2016; Quinn et al. 2009). POPs that have been investigated include organochlorine pesticides (OCPs), polybrominated diphenyl ethers (PBDEs), polychlorinated biphenyls (PCBs), and metals in bird eggs (Bouwman et al. 2008; Chokwe et al. 2015; Polder et al. 2008; Van der Schyff et al. 2016).

Organochlorine pesticides can bioaccumulate in lipid tissue and are resistant to degradation (Newman 2015). In many African countries, the current and historical use of dichlorodiphenyltrichloroethane (DDT) in controlling diseases and pests led to unintentional consequences such as eggshell thinning in many bird species (Bitman et al. 1970; Holm et al. 2006; Lundholm 1997) and human health effects (Bornman et al. 2010). However, less literature is available on other main groups of POPs in bird eggs from South Africa including perfluorooctane sulfonate (PFOS), polychlorinated dibenzofurans and dibenzo-p-dioxins (PCDD/Fs), and PCBs (non-dioxin –like PCBs (NDL-PCBs) and dioxin-like PCB (DL-PCBs)).

Due to the chemical properties (Wania and Mackay 1995) and the effects of POPs, many of these compounds are banned or severely restricted (Stockholm Convention 2016a). Halogenated compounds, such as the chlorinated and brominated compounds, tend to be lipophilic and bioaccumulate in lipid tissues. The DL-PCBs (those PCBs that have chlorine atoms in the non-ortho position) and PCDD/Fs specifically mediate their toxicity via the aryl-hydrocarbon receptor (AhR; Mandal 2005). However, the fluorinated compounds that are also halogenated have both lipophilic and hydrophilic moieties (Newman 2015) which allow them to be distributed by blood to various organs such as the liver, kidneys, and lungs, among others (Kwiatkowski et al. 2021). These compounds cause peroxisomal proliferation, increased activity of lipid and xenobiotic metabolising enzymes (Newman 2015). Residues in the environment reflect current and historical production and use of these compounds (Orisakwe et al. 2019).

The bird egg is a good matrix for environmental monitoring of pollution (Medvedev and Markove 1995; Lebedev et al. 1998). They have a fairly consistent composition, decompose slowly, are easy to handle, and can be randomly sampled in a cost-effective manner. Furthermore, eggs represent the pollutant uptake by the female bird before the egg is laid, while giving insight into the effects, these compounds have on both the female bird and in the developing egg (Braune 2007; van den Steen et al. 2006). In addition, embryonic and foetal development is more sensitive to POPs than in adults since exposure prior or during organ development may have greater consequences than after (Caralson and Duby 1973). Moreover, many bird species are widely distributed over multiple continents and provide opportunities for continental comparison (Lesch et al. 2023).

Both aquatic and terrestrial birds have been used as pollution indicators (Aurigi et al. 2000; Bouwman et al. 2019, 2021; Eljarrat et al. 2019). Elevated PFOS concentrations can lead to endocrine disruption (Jensen and Leffers., 2008) and organ dysfunction, especialli the liver (Hoff et al. 2005). PCBs can cause reproductive abnormalities and lead to developmental effects (Barron et al. 1995). At elevated concentrations, PBDEs cause behavioural and growth abnormalities in the American kestrel (*Falco sparverius*; Fernie et al. 2006 and 2008).

Knowledge of POPs of the Stockholm convention on persistent organic pollutants (SCPOPs) in Southern Africa is restricted. The current study was carried out under the auspices of the Orange-Senqu River Commission’s (ORASECOM) 2010 Joint Basin Survey on POPs in the OSRB as part of the transboundary diagnostic analysis of the OSRB that also evaluated all POPs listed at that time. The aims of this study were therefore to investigate the concentrations of 22 POPs, as listed in the SCPOPs in 2010, in wild bird eggs from the Vaal River. Based on the data, we will identify pollution hotspots, assess the hazard that the compound concentrations may have to the developing embryo and compare the concentrations with concentration reported in literature. Additionally, we determined the concentrations between different species, feeding guilds, and habitat preferences. Lastly, we evaluated the relationship between egg size and POPs concentrations. This study, based on 2010 data, serves as a baseline for future work, identify compounds that need no further attention, but specifically highlight compounds and compound classes of concern that would also inform other studies in Southern Africa. As far as we know, this is the only study that analysed all 2010 POPs in bird eggs on a large geographic scale.

## Materials and Methods

### Bird Egg Sampling

#### Bird Egg Sampling Locations and Descriptions

The necessary provincial permits and the appropriate ethical approvals (NWU-00055–07-S3 and NWU-00594–19-A9) were obtained. Wild bird eggs were collected from four breeding colonies in the OSRB, a 192 000 km^2^ catchment during the breeding season (October to February) 2009/10 (Fig. 1). Efforts to date have recorded 154 heronries in South Africa (Harebottle 2019), although this number is an underestimation. The four selected breeding colonies were located during aerial surveys. The Potchefstroom colony location is near the Mooi River and is closed to residential properties and a golf course. The colony at Barbers Pan (a Ramsar site) is in a bird sanctuary with no town or city nearby. The Bloemhof Dam colony was on Snake Island. The Eldorado Park colony is within a suburb in a highly industrialised region of Gauteng province. Eggs from nine species were sampled: Grey Heron (*Ardea cinerea*), African Darter (*Anhinga rufa*), Glossy Ibis (*Ardea melanocephala*), Great White Egret (*Ardea alba*), Reed Cormorant (*Microcarbo africanus*), African Sacred Ibis (*Threskiornis aethiopicus*), Little Egret (*Egretta garzetta*), Cattle Egret (*Bubulcus ibis*), and Glossy Ibis (*Plegadis falcinellus*). General distributions and descriptions, habitat preferences, breeding behaviour, diet, and egg descriptions are summarised in Table S1.

#### Egg Sampling Effort

Eggs were sampled from nests by either climbing trees using rock-climbing gear or using ladders. Although efforts were made to collect eggs of the same species at all sites, this was not possible. Eggs were wrapped in pre-washed foil, labelled, carefully stored in thick egg cartons, and transported to the laboratory where they were photographed before being frozen at -24ºC until sample preparation. Eggs were analysed within 6 months of collection. On the day of sample preparation, selected eggs were measured and pooled per species and location as presented in Table 1. Egg contents were ultrasonically homogenised. Samples of the 16 pools were sent with the necessary permits to Oëkometric GmbH—The Bayreuth Institute of Environmental Research, in Germany. This is an accredited POPs laboratory. Coordinates of sampling locations, the closest water source, and analytical pool numbers are presented in Table 1.

**Table 1:** Summary of the wild bird species from which eggs were collected at each location, along with the GPS coordinates, closest river, pool number, number of eggs per pool (n), habitat and feeding guilds according to Hockey et al. (2005), and mean egg mass (g)

Location	Longitude	Latitude	River	Pool no	n	Common name	Scientific name	Habitat guild	Feeding guild	Egg mass
Barbers Pan	25,57	−266	Harts River	1	6	Grey Heron	*Ardea cinerea*	Aquatic	Large aquatic predator	61
				5	5	African Darter	*Anhinga rufa*	Aquatic	Large aquatic predator	37
				14	5	Black-headed Heron	*Ardea melanocephala*	Terrestrial	Terrestrial insectivore	60
Bloemhof Dam	2564	−277	Vaal River	2	3	Great White Egret	*Ardea alba*	Aquatic	Large aquatic predator	61
				3	5	Grey Heron	*Ardea cinerea*	Aquatic	Large aquatic predator	61
				6	3	African Darter	*Anhinga rufa*	Aquatic	Large aquatic predator	37
				8	5	Reed Cormorant	*Microcarbo africanus*	Aquatic	Large aquatic predator	21
				10	6	African Sacred Ibis	*Threskiornis aethiopicus*	Wetland	Scavenger	62
				11	4	Little Egret	*Egretta garzetta*	Aquatic	Small aquatic predator	28
				16	6	Cattle Egret	*Bubulcus ibis*	Terrestrial	Terrestrial insectivore	27
Eldorado Park	2788	−263	Klip River	4	5	African Sacred Ibis	*Threskiornis aethiopicus*	Wetland	Scavenger	62
Potchefstroom	2709	−2678	Mooi River	7	5	Reed Cormorant	*Microcarbo africanus*	Aquatic	Large aquatic predator	21
				9	5	Glossy Ibis	*Plegadis falcinellus*	Wetland	Small aquatic predator	34
				12	5	Black-headed Heron	*Ardea melanocephala*	Terrestrial	Terrestrial insectivore	60
				13	4	Black-headed Heron	*Ardea melanocephala*	Terrestrial	Terrestrial insectivore	60
				15	5	Cattle Egret	*Bubulcus ibis*	Terrestrial	Terrestrial insectivore	27

### Chemical Analyses

All samples were analysed within 6 months of collection**.** Laboratory analysis was undertaken by Oëkometric GmbH—The Bayreuth Institute of Environmental Research, in Germany. All POPs analyses were executed with quality assurance and quality control protocols as per ISO/IEC 17025:2005 accreditation that covered, preparation, calibration, extraction, clean-up, measurement, quantification, quality control, concentration calculations, and reporting. Chemical analysis and compounds analysed are presented in Table 2. Laboratory blanks and internal reference material were routinely analysed for quality assurance and QA/QC procedures. Toxic equivalency quotients (TEQs) were calculated according to the WHO (2005), and all are reported as exclusive (van den Berg et al. 2006).

**Table 2:** Summary of the chemical analysis and compounds analysed

PCDD/F	18 PCBs	PBDE classes	PFOS	21 pesticides
PCDD/F	DL-PCB	TetraBDE	Perfluorooctanesulfonic acid (PFOS)	α-Hexachlorocyclohexane (α-HCH)
2,3,7,8-TCDD	PCB 77	PentaBDE		β-Hexachlorocyclohexane (β-HCH)
1,2,3,7,8-PeCDD	PCB 81	HexaBDE		γ-Hexachlorocyclohexane (Lindane)
1,2,3,4,7,8-HxCDD	PCB 126	HeptaBDE		Hexachlorobenzene (HCB)
1,2,3,6,7,8-HxCDD	PCB 169	HexaBB		Heptachlor
1,2,3,7,8,9-HxCDD	PCB 105			Aldrin
1,2,3,4,6,7,8-HpCDD	PCB 114			Dieldrin
OCDD	PCB 118			Endrin
2,3,7,8-TCDF	PCB 123			Heptachloroepoxide
1,2,3,7,8-PeCDF	PCB 156			Chlordane (trans-)
2,3,4,7,8-PeCDF	PCB 157			Chlordane (cis-)
1,2,3,4,7,8-HxCDF	PCB 167			o,p'-DDE
1,2,3,6,7,8-HxCDF				p,p-DDE
1,2,3,7,8,9-HxCDF				o,p'-DDD
2,3,4,6,7,8-HxCDF				p,p-DDD
1,2,3,4,6,7,8-HpCDF				o,p'-DDT
1,2,3,4,7,8,9-HpCDF				p,p-DDT
OCDF				Mirex
				Pentachlorobenzene
				Chlordecone
				Toxaphene
Regulation EC 1883/2006 and EPA 1613 B	Regulation EC 1883/2006, ASU L 00.00–12 and ASU L 00.00–38	Proprietary method and EPA 1614	Proprietary method	Regulation ASU L 00209,.00–12, ASU L 00.00–38 Proprietary method (based on S19 multimethod)
High resolution GC/MS	High resolution GC/MS	High resolution HRGC/HRMS. To minimise the degradation of BDE 209, a short column (15 cm) was used (Agilent DB-5 ms 15 m × 0.25 mm × 0.25 µm) instead of the 30 cm column that were used for the other congeners	Using LC/MS–MS A daily internal lab blank was used as the QA/QC. the lab blanks were routinely analysed (once a week)	High resolution GC/MS
LOQ TCDD/F–HxCDD/F = 0.00005 ng/g wet mass (wm), HpCDD/F = 0.00015 ng/g wm, OCDD/F = 0.0005 ng/g wm)	LOQ DL-PCB 81, 126, 169 = 0.0005 ng/g wm, DL-PCB 77, 105, 114, 123, 156, 157, 167,169 = 0.005 ng/g wm, DL-PCB 118 = 0.050 ng/g wm, NDL-PCB 28, 52, 101, 138, 180 = 0.1 ng/g wm)	LOQ 0.05–0.1 ng/g wm	LOQ = 1 ng/g wm	LOQ = 0.1–2 ng/g wm

### Statistical Analyses and Measuring Unit Conversions.

Descriptive and comparative statistics were performed using GraphPad Prism version 10.2.0. Concentration unit conversions were performed to compare published and current data. The data were received from the laboratory in wet mass (wm). The values reported in parts per million (ppm), parts per billion (ppb), milligrammes per kilogramme (mg / kg), and microgrammes per kilogramme (µg/kg) by other authors were converted to nanogrammeme per gramme (ng/g). The concentration values reported in lipid mass (lm) by other authors were converted to wet mass (wm) (Clatterbuck et al. 2018). We evaluated and compared wet mass (wm)-based data, given that embryo development affects lipid composition more than water content (Herzke et al. 2002; Romanoff 1932). The current data were converted to data based on lipid mass (lm) and are presented in Table S2. The determination of lipids was done gravimetrically.

The ΣPCB value is the total concentration of both DL-PCBs and NDL-PCBs. The PCB TEQ value consists of only DL-PCBs. The logarithmic transformation of the POP classes was regressed against the egg mass. Firstly, Prism compares whether slopes are parallel, calculating a two-tailed p-value. The null hypothesis is that the slopes are identical and therefore parallel. Second, Prism calculates if the Y-intercepts (elevations) for the regression lines are identical. Low p-values signify that the slopes and intercepts are significantly different.

## Results

**Fig. 2:**
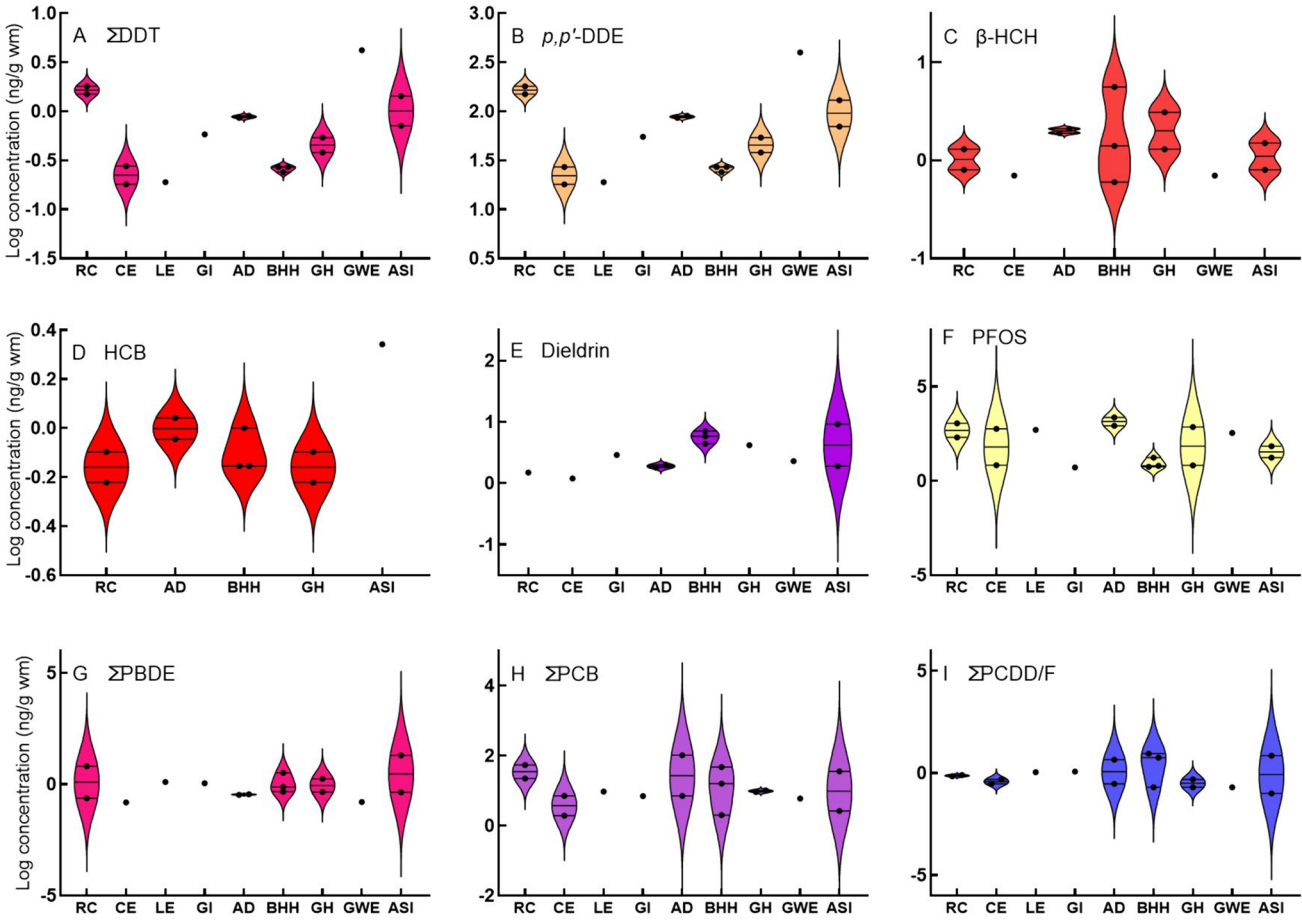
Violin plots (frequency distributions) of log-transformed concentrations of selected compounds quantified in bird eggs regardless of sampling location. Horizontal lines are medians and 25 and 75% quartiles. Species are arranged according to increasing reported mean egg mass. RC = Reed Cormorant, CE = Cattle Egret, LE = Little Egret, GI = Glossy Ibis, AD = African Darter, BHH = Black Headed Heron, GH = Grey Heron, GWE = Great White Egret, and ASI = African Sacred Ibis

**Fig. 3:**
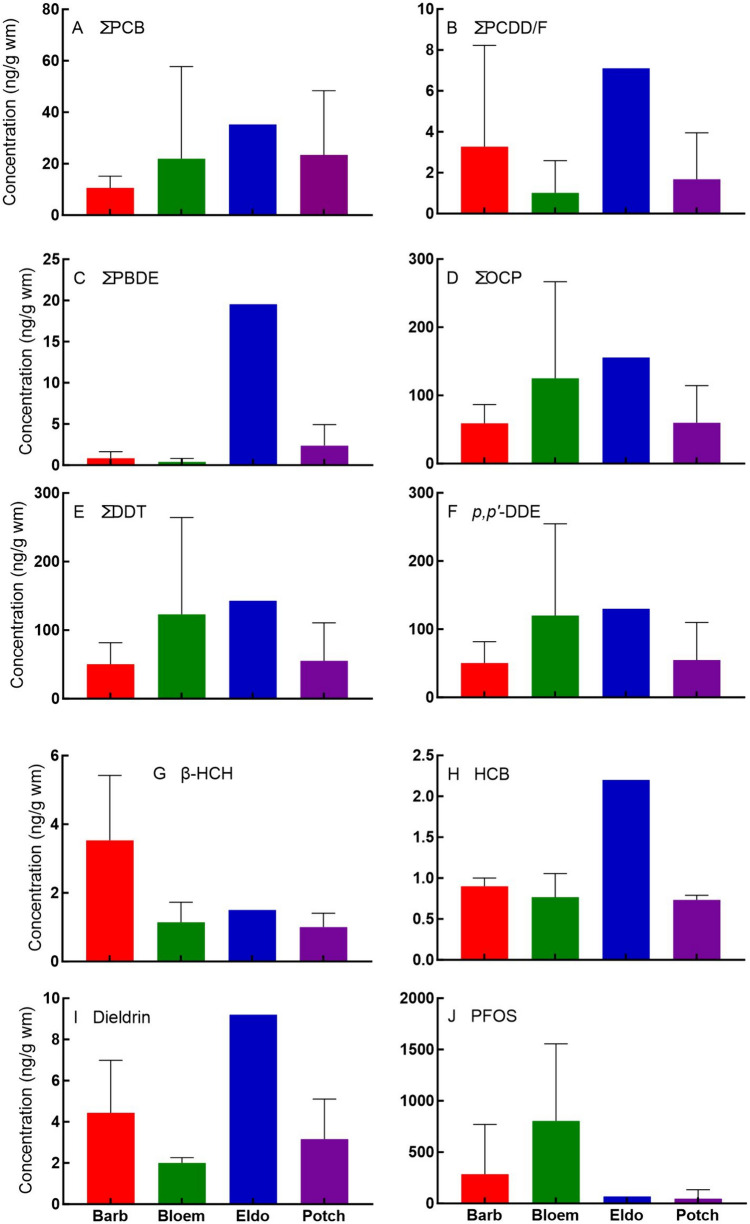
Mean concentrations and standard deviations of selected compounds quantified at each sampling location regardless of species. Barb = Barbers Pan, Bloem = Bloemhof Dam, Eldo = Eldorado Park, and Potch = Potchefstroom

**Fig. 4:**
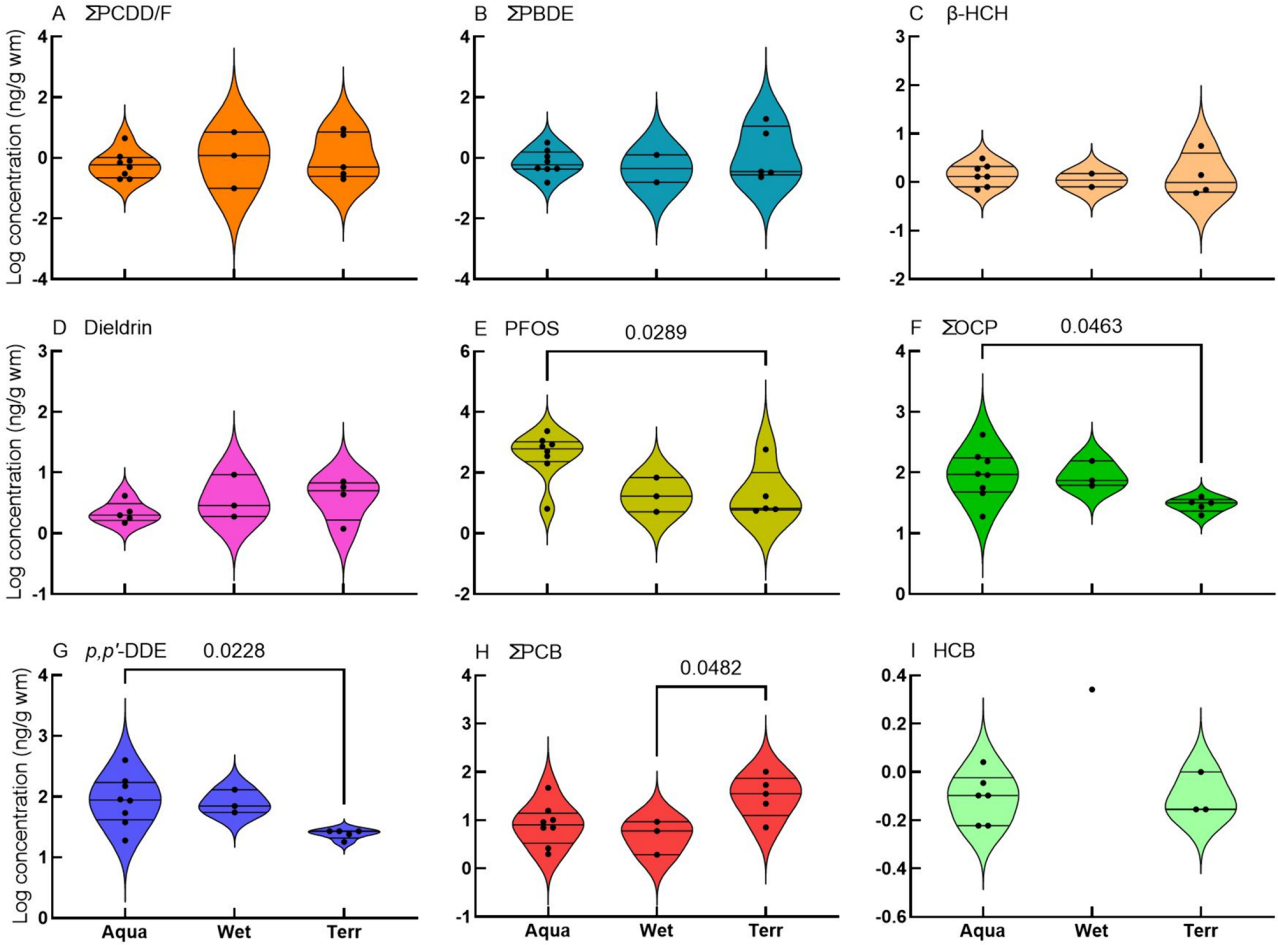
Violin plots (frequency distributions) of log-transformed concentrations of selected compounds quantified in bird eggs according to habitat guilds. Horizontal lines are medians and 25 and 75% quartiles. Habitat guilds are expressed as aquatic, wetland, and terrestrial. Aqua = aquatic, Wet = wetland, and Terr = terrestrial. ANOVA p-values of guilds that were found to be statistically significant different are indicated with brackets. Two-way unpaired t-test was performed for HCB

**Fig. 5:**
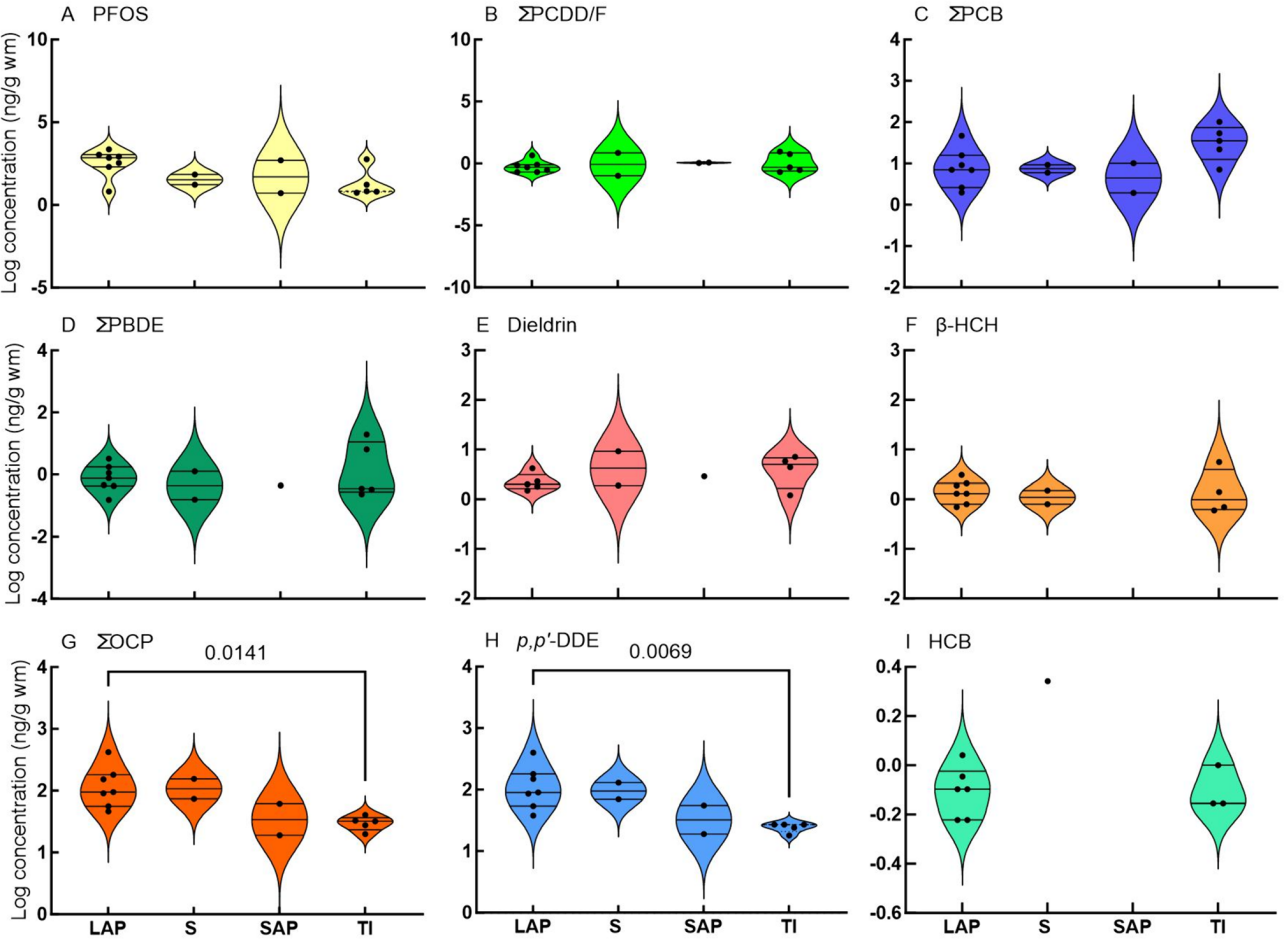
Violin plots (frequency distributions) of concentrations of selected compounds quantified in bird eggs according to feeding guilds. Horizontal lines are medians and 25 and 75% quartiles. Feeding guilds are expressed as LAP = large aquatic predators, S = scavengers, SAP = small aquatic predators, and TI = terrestrial insectivore. ANOVA p-values of guilds that were found to be statistically significant different are indicated with brackets. Two-way unpaired t-test was performed for HCB

**Fig. 6:**
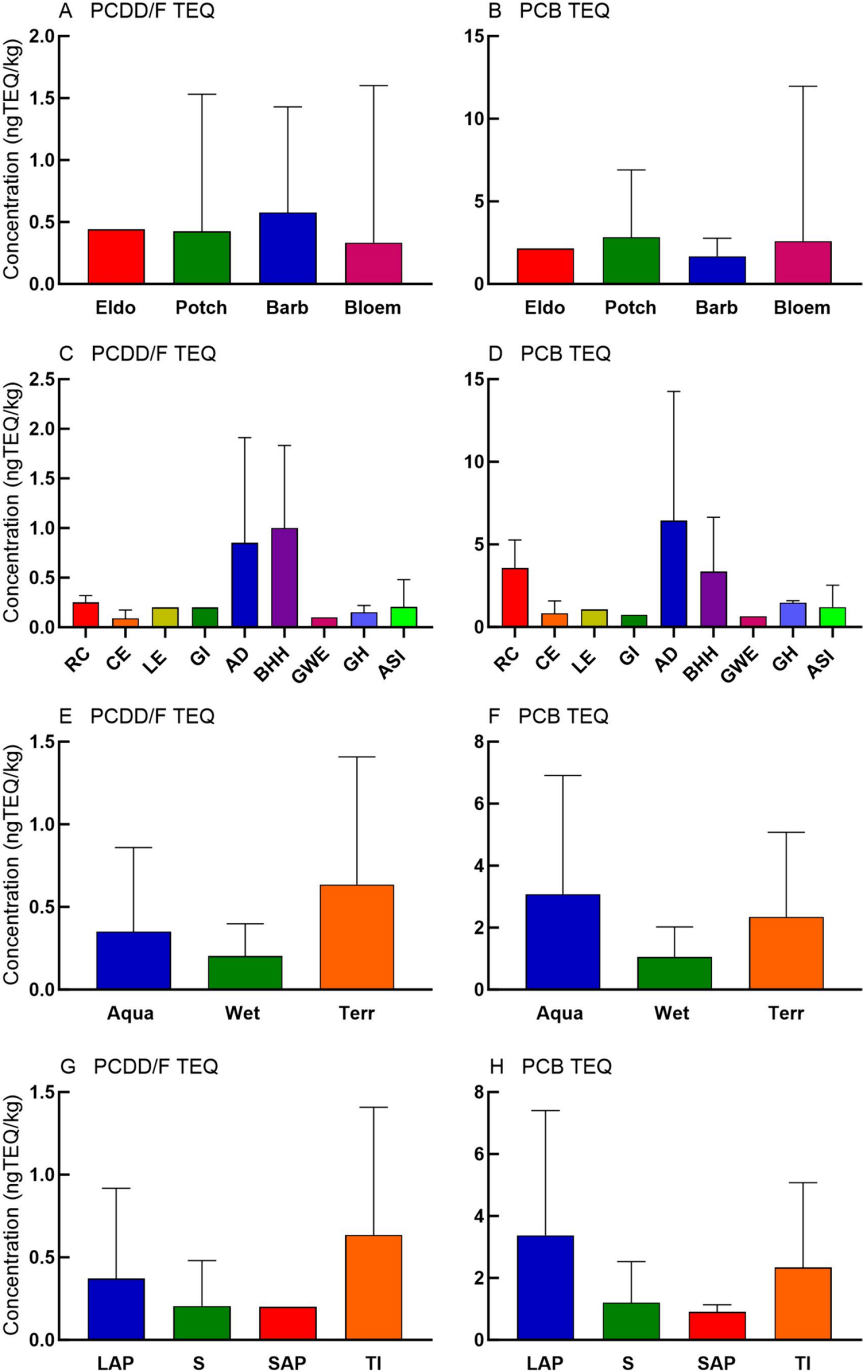
TEQ values in bird eggs. Data are expressed as mean with range. A) PCDD/F TEQ values according to sampling location. B) PCB TEQ values according to sampling location. Sampling locations are expressed as Barb = Barbers Pan, Bloem = Bloemhof Dam, Eldo = Eldorado Park and Potch = Potchefstroom. C) PCDD/F TEQ values according to species. D) PCB TEQ values according to species. Species are arranged according to increasing reported average egg mass. Species are expressed as RC = Reed Cormorant, CE = Cattle Egret, LE = Little Egret, GI = Glossy Ibis, AD = African Darter, BHH = Black Headed Heron, GH = Grey Heron, GWE = Great White Egret, and ASI = African Sacred Ibis. E) PCDD/F TEQ values according to habitat guilds. F) PCB TEQ according to habitat guilds. Habitat guilds as expressed as Aqua = aquatic, Wet = wetland, and Terr = terrestrial. G) PCDD/F TEQ values according to feeding guilds. H) PCB TEQ according to feeding guilds. Feeding guilds are expressed as LAP = large aquatic predators, S = scavengers, SAP = small aquatic predators, and TI = terrestrial insectivore

**Fig. 7:**
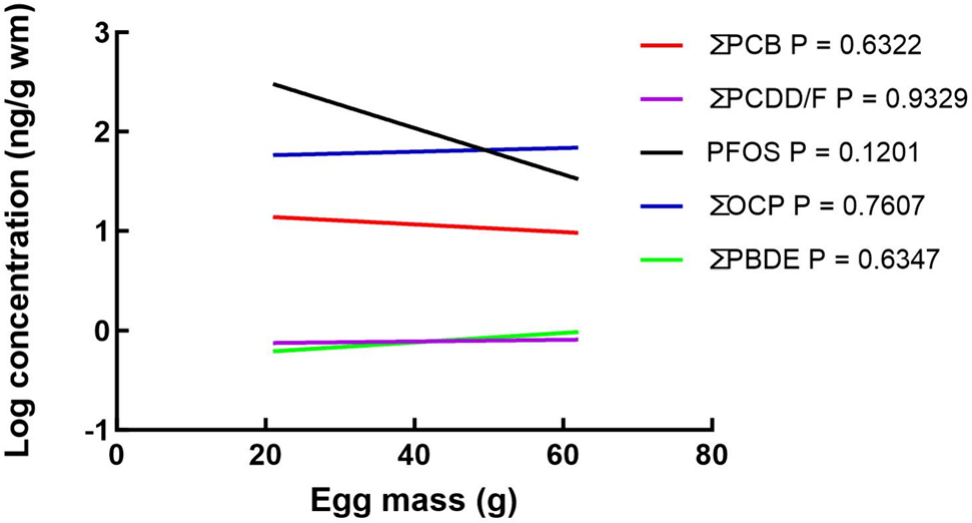
Simple linear regression. Concentrations of compound classes, regardless of species or sample location, regressed against egg mass

Summary results are given in Table 3 and presented in several ways in Figs.2, 3, 4, 5, 6, 7. The concentration quantified of individual congeners can be viewed in the supplementary material in Table S2. And the results converted to lipid mass (lm) are given in Table S3.

**Table 3:** Summary of all quantified concentrations detected in wild bird eggs. All concentrations are expressed in ng/g wet mass (wm) except for the TEQ values which are expressed in ng/kg wm. Values based on lipid mass (lm) are provided in Table S3

			ng/g wm														ng/kg wm	
Species	Site	Pool no	PFOS	ΣPBDE	β-HCH	HCB	Dieldrin	p*p,p'*-DDD	*p,p'*-DDT	*p,p'*-DDE	ΣDDT	ΣOCP	Dl- PCB*	NDL- PCB**	ΣPCB	∑PCDD/F	WHO-TE 2005: Exclusive	
																	WHO PCB TEQ	WHO PCDD/F TEQ
Grey Heron	Barbers Pan	1	7	2	3	1	4			38	38	46	1	8	9	1	1	0.2
Great White Egret	Bloemhof Dam	2	350	0.2	1		2	8	12	400	420	420	1	5	6	0.2	0.7	0.1
Grey Heron	Bloemhof Dam	3	720	0.4	1	1				54	54	56	1	9	10	0.2	2	0.1
African Sacred Ibis	Eldorado Park	4	69	20	2	2	9		10	130	140	160	3	33	35	7	2	0.4
African Darter	Barbers Pan	5	850	0.3	2	1	2	3		86	86	91	1	6	7	0.3	1	0.1
African Darter	Bloemhof Dam	6	2300	0.4	2	1	2			90	90	95	14	88	100	5	12	2
Reed Cormorant	Potchefstroom	7	200	6	1	1	2			150	150	150	9	45	54	1	5	0.2
Reed Cormorant	Bloemhof Dam	8	1100	0.2	1	1				180	180	180	4	18	22	1	2	0.3
Glossy Ibis	Potchefstroom	9	5	1			3	1	3	55	58	61	1	6	7	1	1	0.2
African Sacred Ibis	Bloemhof Dam	10	17	0.4	1		2		1	70	71	74	0.3	2	3	0,1	0.3	0.01
Little Egret	Bloemhof Dam	11	500	1						19	19	19	1	8	9	1	1	0.2
Black-headed Heron	Potchefstroom	12	6	1	1	1	4			27	27	33	0.2	2	2	0.2	0.4	0.04
Black-headed Heron	Potchefstroom	13	17	3	1	1	6			24	24	32	4	43	47	6	7	2
Black-headed Heron	Barbers Pan	14	6	0.5	6	1	7			27	27	41	2	14	16	9	3	1
Cattle Egret	Potchefstroom	15	7	0.2	1		1			18	18	20	1	6	7	1	1	0.2
Cattle Egret	Bloemhof Dam	16	580						1	27	28	28	0.3	2	2	0.3	0.3	0.03

^*^Dl- Dioxin like

^**^ NDL-Non-dioxin like

### Bird Egg Concentrations

Organochlorine compounds such as α-HCH, lindane, heptachlor, aldrin, endrin, heptachloroepoxide, chlordane (trans- and cis -), mirex, pentachlorobenzene, chlordecone, toxaphene, *o,p'*-DDE, *o,p'*-DDD, and *o,p'*-DDT were detected in all eggs, but at concentrations below the LOQ.

The highest ΣOCP concentration was quantified in eggs of Great White Egret eggs (423 ng/g wm) from Bloemhof Dam, primarily as a result of the high *p,p’*-DDE (400 ng/g wm) (Fig. 2A and B; Table 3). This egg pool (Table 1) had double the ΣOCP concentration than Reed Cormorant eggs from Bloemhof Dam (180 ng/g wm), Potchefstroom (150 ng/g wm), and an order of magnitude greater than the African Sacred Ibis egg pool from Eldorado Park (19 ng/g wm) (Table 3). Most of the ΣOCP concentrations were composed of *p,p’*-DDE. However, other OCPs were also quantified in some eggs (Table 3). The highest β-HCH concentration was in Black-headed Heron eggs (6 ng/g wm) from Barbers Pan (Fig. 2C; Table 3). The highest HCB (2 ng/g wm) (Fig. 2D) and dieldrin (9 ng/g wm) (Fig. 2E) concentrations were in African Sacred Ibis eggs from Eldorado Park.

The highest PFOS concentrations were quantified in African Darter eggs (2300 ng/g wm) and Reed Cormorant eggs (1100 ng/g wm) from Bloemhof Dam (Fig. 2E). PFOS was also the dominant compound in most species, except Great White Egret, African Sacred Ibis, Black-headed Heron, and Glossy Ibis where ΣOCPs dominated (Table 3). African Sacred Ibis eggs from Eldorado Park had the highest ΣPBDE concentrations (19 ng/g wm), followed by Reed Cormorant eggs from Potchefstroom (Fig. 2G; Table 3). The highest ΣPCB concentration in any pool was in African Darter eggs (100 ng/g wm) from Bloemhof Dam followed by Reed Cormorant eggs (54 ng/g wm) from Potchefstroom (Figure 2H; Table 3). ΣPCDD/F concentrations were highest in Black-headed Heron eggs (9 ng/g wm) from Barbers Pan, followed by African Sacred Ibis eggs from Eldorado Park (7 ng/g wm) (Fig. 2I).

Irrespective of species, the highest mean ΣPCB, ΣPCDD/F, ΣPBDE, ΣOCP, and ΣDDT were found in eggs from Eldorado Park (Fig. 3A–E). In addition, all OCPs were higher at Eldorado Park, except β-HCH which was higher at Barbers Pan (Fig. 3F–I). Bloemhof Dam had the highest mean PFOS concentration, followed by Barbers Pan (Fig. 3J).

### Guilds

The species were grouped according to habitat guilds: aquatic, terrestrial, and wetland (Table 1; Fig. 4). There were no significant differences between habitat guilds (one-way ANOVA, Tukey’s multiple comparisons) for ΣPCDD/F, ΣPBDE, β-HCH, and dieldrin (Fig. 4A–D). There were statistically significant differences between aquatic and terrestrial habitat guilds for PFOS, ΣOCP, and *p,p’*-DDE (Fig. 4E–G), and between terrestrial and wetland habitat guilds for ΣPCB (Fig. 4I). Since only one data point was available for HCB in the wetland guild, we performed a two-way, unpaired t-test between terrestrial and aquatic eggs which was not significantly different (Fig. 4H).

We grouped all species according to their feeding guilds: large aquatic predators (LAP), small aquatic predators (SAP), scavengers (S), and terrestrial insectivores (TI) (Table 1). There were no significant differences between feeding guilds (one-way ANOVA, Tukey’s multiple comparison) for PFOS, ΣPCDD/F, ΣPCB, ΣPBDE, dieldrin, and β-HCH (Fig. 5A-F). A statistically significant difference was found between large aquatic predators and terrestrial insectivores for ΣOCP and *p,p’*-DDE (Fig. 5G and H). We performed two-way, unpaired, *t-*tests for HCB and found no statistically significant differences (Fig. 5I).

### TEQ

Mean PCDD/F TEQ values of bird eggs were highest at Barbers Pan followed by Potchefstroom (Fig. 6A), although the highest PCDD/F TEQ value was from eggs collected at Bloemhof Dam (1.6 ngTEQ/kg wm) (Table 3). PCB TEQ values in bird eggs were highest at Bloemhof Dam (12 ngTEQ/kg wm) followed by Potchefstroom (Fig. 6B). PCDD/F and PCB TEQ values were highest in African Darter (12 ngTEQ/kg wm) and Black-headed Heron (7 ngTEQ/kg wm) eggs, followed by Reed Cormorant eggs (5 ngTEQ/kg wm) (Fig. 6C and D). PCDD/F TEQ values were highest in terrestrial species (Fig. 6E) while PCB TEQ values dominated in aquatic habitat guild eggs (Fig. 6F). PCDD/F TEQ values were highest in terrestrial insectivores (Fig. 6G) while PCB TEQ values were highest in large aquatic predators (Fig. 6H). The PCB TEQs were higher in all species and at all sites compared to the PCDD/F TEQ values (Table 3).

### Influence of Egg Mass

We used linear regression to investigate the association of compound classes with egg mass (Fig. 7). None of the slopes were significantly different from the X-axis. We also tested whether slopes and intercepts (vertical distances between the Y-intercepts of each slope – Y-intercepts indicates where the regression slopes meet the Y-axes) were significantly different from each other (Prism uses a method similar to analysis of covariance (ANCOVA)). The slopes themselves were not significantly different from zero or each other (*p* = 0.2773). There was, however, a significant difference between Y-intercepts (*p* < 0.0001, ANOVA; *p* < 0.0001, Brown-Forsythe test; *p* < 0.0001, Bartlett’s test).

## Discussion

### Bird Egg Concentrations

#### Feeding Guilds

With eggs of nine species of birds collected from four locations and measured for 22 POPs, the current study is a multi-species analyses investigating the pollution load of both aquatic and terrestrial birds. POP concentrations differed greatly between species, sites, habitat groups, and feeding groups (Table 3 and Figs. 2, 3, 4, 5). This was as expected since the sites and species were collected over a large area where breeding colonies were available. The locations of the active breeding colonies at the time of sampling were found via aerial reconnaissance for this specific purpose. However, there are apparent patterns based on guilds and localities close to sources, with some exceptions.

Eggs from species that occupy high-trophic levels had higher concentrations of PFOS and ΣOCP concentrations, while species that feed on insects had lower concentrations (Table 3 and 4 and Fig. 5). This was reflected in habitat guilds where aquatic species had higher PFOS and ΣOCP concentrations (Fig. 4). This pattern was also noted by Eriksson et al. (2016) who found higher concentrations of PFAS in eggs from aquatic species compared to terrestrial species. The ΣOCP concentrations were dominated by *p,p’*-DDE, which is in agreement with others (Bouwman et al. 2008; Venugopal et al. 2020). Although we did not observe any pattern regarding PBDE concentrations in guilds, She et al. (2008) did find higher ΣPBDE concentrations in eggs of piscivorous birds compared with omnivorous species in the USA.

**Table 4:** Mean concentrations of compound groups (ng/g wm) in wild bird eggs reported by various authors. The country, location, and year the samples were collected and are shown. All reported concentrations were converted to ng/g wm

Species	Country	Year sampled	Location	ΣPCB_?_	ΣPCB^#^	∑PCDD/F	PFOS	∑BDE_?_	PBDE	∑OCP	Reference
*Grey Heron (Ardea cinerea)*											
	RSA	2009	Barbers Pan	ΣPCB_6_	9	0.5	7	ΣBDE_5_	2	46	This study
	RSA	2009	Bloemhof Dam	ΣPCB_6_	10	0.2	725	ΣBDE_5_	0.5	56	This study
	Turkey*	2009	SÖKE	ΣPCB_7_	70					1100	Kocagöz et al. 2014
	RSA*	2009	Nandoni Dam	ΣPCB_20_	38					14,276	Bouwman et al. 2013
	Creece	2004	Lake Kerkini	ΣPCB_7_	64						Antoniadou et al. 2007
	Spain	2010–17	Castrejón reservoir					ΣBDE_8_	119		Eljarrat et al. 2019
	Romania*	1997	Danube Delta	ΣPCB_6_	620					2321	Aurigi et al. 2000
	Greece*	2004	Lake Kerkini							255	Goutner et al. 2012
	France	1991	Reserve Naturelle de Grandlieu	ΣPCB_1_	1280					745	de Cruz et al. 1997
*Blue Heron (Ardea herodias)*											
	Canada	1979	Quebec	ΣPCB_?_	7880					5266	Laporte 1982
	Canada^2,3^	1995	Columbia River, Bachelor island	ΣPCB_3_	615	0.04				0.4	Thomas and Anthony 1999
	Canada (2 and 3)	1994	Columbia River, Ross island	ΣPCB_3_	3454					2	Thomas and Anthony 1999
	USA	1993	Indiana Dunes				245	ΣBDE_6_	347		Custer et al. 2009
	Canada (3)	2002	Fraser River Estuary					ΣBDE_6_	457		Miller et al. 2015
	Canada (2)	1989	University of Brithish Columbia			0.4					Elliott et al. 2001
	Canada (2)	1991	Victoria			0.1					Elliott et al. 2001
	Canada (2)	1987	Crofton			1					Elliott et al. 2001
	USA (2)	1993	Mississippi river / Pig's Eye				940	ΣBDE_7_	142		Custer et al. 2010
*Black-headed Heron (Ardea melanocephala)*											
	RSA	2009	Potchefstroom	ΣPCB_6_	2	0.2	6	ΣBDE_5_	1	33	This study
	RSA	2009	Potchefstroom	ΣPCB_7_	47	6	17	ΣBDE_5_	3	32	This study
	RSA	2009	Barbers Pan	ΣPCB_8_	16	9	6	ΣBDE_5_	0.5	41	This study
*Night Heron (Genera: Nycticora, Nyctanass, and Gorsachius)*											
	Romania*	1997	Danube Delta	ΣPCB_6_	127					1689	Aurigi et al. 2000
	Israel	1975	Coastal plain	ΣPCB_?_	770					1620	Perry et al. 1990
	USA	1996	Alexander island	ΣPCB_18_	2100	0.2				460	Frank et al. 2001
	Italy	1994	Riserva Naturale Garzaia di Villarasca	ΣPCB_?_	40					200	Fasola et al. 1998
	Creece	2004	Lake Kerkini	ΣPCB_7_	26						Antoniadou et al. 2007
	Hong Kong	2000	A Chau			0.1					Wang et al. 2012
	Hong Kong	2000	Mai Po Village	ΣPCB_?_	230					704	Connell et al. 2003
	China	2004	Xiamen				123				Wang et al. 2008
	Hong Kong	2006	A Chau				115				Wang et al. 2008
	Spain	2010–17	Castrejón reservoir					ΣBDE_8_	15		Eljarrat et al. 2019
	Greece*	2004	Lake Kerkini							172	Goutner et al. 2012
	Hong Kong*	2006	A Chau	ΣPCB_?_	55					186	Wang et al. 2011
*Purple Heron (Ardea purpurea)*											
	Spain	2010–17	Castrejón reservoir					ΣBDE_8_	43		Eljarrat et al. 2019
*Reed Cormorant (Microcarbo africanus)*											
	RSA	2009	Potchefstroom	ΣPCB_6_	54	1	201	ΣBDE_5_	6	154	This study
	RSA	2009	Bloemhof Dam	ΣPCB_6_	22	1	1120	ΣBDE_5_	0.2	181	This study
	RSA	2004/5	Vaal River	ΣPCB_34_	110					308	Bouwman et al. 2008
	RSA*	2004/5	Parys	ΣPCB_34_	165			ΣBDE_8_	1	449	Polder et al. 2008
*Great Cormorant (Phalacrocorax carbo)*											
	Netherlands	1988/9	Rhine and Meuse rivers	ΣPCB_6_	1583					5318	Dirksen et al. 1995
	Sweden (1)	2007–9	Lake Vänern				552				Nordén et al. 2013
	Germany	2009	Baltic sea / Heuwiese				90				Rüsdel et al., 2011
	Germany	2009	Elbe estuary/ Haseldorf				540				Rüsdel et al., 2011
	Greece*	2004	Lake Kerkini							355	Goutner et al. 2012
	Creece	2004	Lake Kerkini	ΣPCB_7_	60						Antoniadou et al. 2007
*Neotropic Cormorant (Nannopterum brasilianum)*											
	USA	1996	Alexander island	ΣPCB_18_	5720	0.1				1364	Frank et al. 2001
	USA	1996	San Bernard Wildlife refuge	ΣPCB_18_	404					493	Frank et al. 2001
	USA	1996	Smith Point	ΣPCB_18_	1640	0.01				213	Frank et al. 2001
	USA	1996	Vingt-et-un	ΣPCB_18_	3140	0.01				423	Frank et al. 2001
*White-breasted Cormorant (Phalacrocorax lucidus)*											
	RSA	2013	KwaZulu-Natal							600	Bouwman et al. 2019
*Double-crested Cormorant (Nannopterum auritum)*											
	Canada (5)	1973	Mandarte island			0.1					Harris et al. 2003
	Canada (5)	1998	Mandarte island			0.04					Harris et al. 2003
	Canada (5)	1987	Crofton			1					Harris et al. 2003
	Canada (5)	1997	Crofton			0.1					Harris et al. 2003
	Canada (3)	1994	Mandarte island					ΣBDE_9_	385		Miller et al. 2015
*African Darter (Anhinga rufa)*											
	RSA	2009	Barbers Pan	ΣPCB_6_	7	0.3	846	ΣBDE_5_	0.3	91	This study
	RSA	2009	Bloemhof Dam	ΣPCB_6_	102	5	2330	ΣBDE_5_	0.4	95	This study
	RSA	2004/5	Vaal River	ΣPCB_34_	300					370	Bouwman et al. 2008
	RSA*	2004/5	Parys	ΣPCB_34_	314			ΣBDE_8_	1	398	Polder et al. 2008
	RSA	2008/9	Gauteng/ Free state	ΣPCB_34_	310			ΣBFR_11_	8	590	Bouwman et al. 2021
	RSA*	2008/9	Kempton Park/ Parys					ΣBDE_9_	11		Quinn et al. 2020
*Great White Egret (Ardea alba)*											
	RSA	2009	Bloemhof Dam	ΣPCB_6_	6	0.2	352	ΣBDE_5_	0.2	423	This study
	Romania*	1997	Danube Delta	ΣPCB_6_	740					4658	Aurigi et al. 2000
	Hong Kong*	2006	A Chau	ΣPCB_?_	126					1059	Wang et al. 2011
	USA	1996	Alexander island	ΣPCB_18_	1510	0.1				379	Frank et al. 2001
*Little Egret (Egretta garzetta)*											
	RSA	2009	Bloemhof Dam	ΣPCB_6_	9	1	505	ΣBDE_5_	1	19	This study
	Spain	2006	Aiguabarreig	ΣPCB_7_	230					277	Huertas et al. 2016
	France	1996	Rhône delta	ΣPCB_12_	3305					123	Berny et al. 2002
	Creece	2004	Lake Kerkini	ΣPCB_7_	18						Antoniadou et al. 2007
	Hong Kong	2000	Mai Po Village	ΣPCB_?_	960					2440	Connell et al. 2003
	Hong Kong*	2000	Mai Po Village	ΣPCB_?_	288					417	Wang et al. 2011
	Italy	1993/4	Riserva Naturale Garzaia di Villarasca	ΣPCB_?_	77					249	Fasola et al. 1998
	China	2004	Xiamen				70				Wang et al. 2008
	RSA	2013	KZN							500	Bouwman et al. 2019
	Romania* (4)	1997	Danube Delta	ΣPCB_6_	546					12,448	Aurigi et al. 2000
	Israel	1975	Coastal plain	ΣPCB_?_	540					1610	Perry et al. 1990
	Greece*	2004	Lake Kerkini							103	Goutner et al. 2012
*Cattle Egret (Bubulcus ibis)*											
	RSA	2009	Potchefstroom	ΣPCB_6_	7	0.5	7	ΣBDE_5_	0.2	20	This study
	RSA	2009	Bloemhof Dam	ΣPCB_6_	2	0.3	579	ΣBDE_5_	0	28	This study
	RSA*	2009	Elim	ΣPCB_20_	9					26	Bouwman et al. 2013
	RSA*	2009	Tshakhuma Dam	ΣPCB_20_	5					104	Bouwman et al. 2013
	RSA*	2009	Xikundu dam	ΣPCB_20_	6					307	Bouwman et al. 2013
	RSA	2004/5	Baberspan	ΣPCB_34_	4					28	Bouwman et al. 2008
	RSA	2004/5	Vaal River	ΣPCB_34_	8					28	Bouwman et al. 2008
	RSA*	2004/5	Barberspan	ΣPCB_34_	3			ΣBDE_8_	0.1	23	Polder et al. 2008
	RSA*	2004/5	Parys	ΣPCB_34_	8			ΣBDE_8_	0.3	30	Polder et al. 2008
	RSA	2008/9	Gauteng/ Free state	ΣPCB_34_	16			ΣBFR_11_	4	21	Bouwman et al. 2021
	Spain	2006	Aiguabarreig	ΣPCB_7_	51					49	Huertas et al. 2016
	China*	2000	Tai Lake							56	Dong et al. 2004
	Israel	1975	Coastal plain							620	Perry et al. 1990
	Hong Kong	2000	Mai Po Village			0.04					Wang et al. 2012
	RSA*	2008/9	Soweto/Parys/Sasolburg					ΣBDE_9_	5		Quinn et al. 2020
*African Sacred Ibis (Threskiornis aethiopicus)*											
	RSA	2009	Eldorado Park	ΣPCB_6_	35	7	69	ΣBDE_5_	20	156	This study
	RSA	2009	Bloemhof Dam	ΣPCB_6_	3	0.1	17	ΣBDE_5_	0.4	74	This study
	RSA	2004/5	Vaal River	ΣPCB_34_	59					94	Bouwman et al. 2008
	RSA*	2004/5	Parys	ΣPCB_34_	65			ΣBDE_8_	14	91	Polder et al. 2008
	RSA	2008/9	Gauteng/ Free state	ΣPCB_34_	59			ΣBFR_11_	53	56	Bouwman et al. 2021
	RSA*	2008/9	Soweto					ΣBDE_9_	54		Quinn et al. 2020
*Glossy Ibis (Plegadis falcinellus)*											
	RSA	2009	Potchefstroom	ΣPCB_6_	7	1	5	ΣBDE_5_	1	61	This study
	Romania*	1997	Danube Delta	ΣPCB_6_	154					939	Aurigi et al. 2000

All values reported from studies other than the current study were reported in concentration units other than ng/kg and had to be converted. Values had to be converted from dry mass or lipid mass to wet mass. (1) Concentration was expressed as the median and not the mean. (2) Data from more than one location or site were reported, but only the location or site with the highest concentration was used in this Table (3) Data from more than one yearly sample run were reported, but only the year with the highest concentration was used in this Table (4) Concentration is of the egg yolk only. (5) Data from more than 1 year were reported, only selected data were used. # The sum concentration of DL-PCBs and NDL-PCBs as reported by authors

The ΣPCDD/F and ΣPCB concentrations suggest higher availability in terrestrial environments, which is contrary to the patterns found by Bouwman et al. (2021). Higher ΣPCB and ΣPCDD/F concentrations were found in soil rather than sediment (Quinn et al. 2009) from the same industrialised region sampled in this study. PCBs and PCDD/F tend to adhere to organic particles, and concentrations may be greater in terrestrial environments as a result (Quinn et al. 2009). These patterns were not seen in other compound classes, perhaps due to the low concentrations in the environment and small sample sizes. Another possible explanation for the lack of patterns observed may be due to differences in foraging behaviour of species (Harris et al. 2003); some species spend prolonged time near the nesting grounds while others roam over larger areas. In addition, differences in prior individual life histories among colony members can lead to differences in POP concentrations. It would have been insightful to compare the POP concentrations to those found in eggs of herbivorous, granivorous, and omnivorous species from the same sites (Bouwman et al. 2021).

We found few other studies with which to compare our findings. Lopez-Antia et al. (2017) reported no significant differences in PFOS concentrations between three species investigated. However, the PFOS concentration was greater in the more aquatic Mediterranean Gull (*Larus melanocephalus*). This pattern was also observed by Bouwman et al. (2021), who reported higher POP concentrations in species that inhabit aquatic habitats and species that are aquatic predators. Another multi-species analysis showed that eggs from omnivore species bioaccumulate a higher ΣOCP concentration than species that feed on only one specific food source (Venugopal et al. 2020).

#### Locations

The eggs of all species collected at Bloemhof Dam, except African Sacred Ibis, had the highest concentrations of PFOS in this survey (Table 3; Fig. 3). This suggests high concentrations of environmentally available PFOS in this region. The African Sacred Ibis eggs from Eldorado Park, in contrast with the other POPs classes (Fig. 3), had higher PFOS concentrations than those of the same species from Bloemhof Dam. Birds from industrialised areas are likely to be exposed to higher POP concentrations than rural birds (Elliott et al. 2015) which may explain the difference in PFOS concentrations between the Eldorado Park and Bloemhof Dam for the African Sacred Ibis (a scavenger, Table S1). Mean concentrations for all other compound classes, except PFOS and β-HCH, were highest at Eldorado Park, located in the highly industrialised Gauteng. Unfortunately, eggs from other species, apart from African Sacred Ibis, were not available in Eldorado Park at the time of sampling, complicating interpretation. A more detailed discussion on sources follows in Sect. "Hotspot identification".

#### Egg Mass

It could be argued that larger birds with larger eggs would eat larger prey from higher trophic levels. This would reflect in larger concentrations of POPs in their eggs. However, Bouwman et al. (2021) found no such effect, even when including eggs from a granivore trophic level such as sparrows (small eggs at ca. 2 g) and high-trophic level African Darters and herons with large eggs (ca. 60 g). For POPs classes such as ΣDDTs, ΣPCBs, and ΣBDEs, there were no associations (linear regressions) of any POP class concentrations with egg mass (Fig. 7), despite orders of magnitude differences in compound class concentrations as signified by the y-intercepts (Fig. 7) and Table 3. Although the eggs of the current study were from birds from a generally high-trophic level, we also found no association of POPs class concentrations such as DDTs and PCBs with egg mass, including for the first time PFOS and ΣPCDD/Fs (Fig. 7). This phenomenon remains difficult to explain.

At higher concentrations of DDT and chlordane’s, eggs of Glaucous gulls (*Larus hyperboreus*) were smaller (Verboven et al. 2009). Verboven et al. (2009) include endocrine disruption, direct toxic effects, poor body condition, and food availability as contributing causes causing smaller eggs. DDT also causes thinner eggshells (Findholt 1984; Peakall 1993), suggesting that eggs with thinner shells weigh less per volume of egg. However, reverse causality should also be considered. During the formation of eggs, those that eventually become lighter may have received proportionally more POPs deposited before the shell is formed. However, arguing this phenomenon across multiple species ranging in egg masses between 21 and 62 g would be difficult. Having observed this phenomenon twice (here, and in Bouwman et al. 2021) with POPs analyses done by two different laboratories invites further investigation.

### Comparisons With International Data

Many studies have reported POP concentrations in bird eggs. For the current study, we selected articles that used the same species or similar species for comparison (Table 4). The majority of published literature on POP concentrations in eggs primarily focused on PCBs and OCPs, especially DDT and its metabolites. ΣOCP concentrations in all species were generally lower compared to international studies. Little Egret eggs had ΣOCP concentrations two orders of magnitude lower than eggs from Spain (Huertas et al. 2016), and up to three orders of magnitude lower than eggs from France (Berny et al. 2002) and Romania (Aurigi et al. 2000).

∑PBDE concentrations in eggs of the present study were low compared with international data (Table 4). Concentrations quantified in Grey Heron eggs from Barbers Pan were two orders of magnitude lower than ΣPBDE concentrations in eggs from Spain, Canada, and the USA (Table 4; Custer et al. 2009; Eljarret et al. 2019; Miller et al. 2015). PFOS concentrations in eggs from the current study were generally lower, or of the same order of magnitude, than reported from other regions, except for eggs from Bloemhof Dam (Table 4). Night Heron (*Nycticorax nycticorax*) eggs from China had lower PFOS concentrations than Grey Heron eggs from Bloemhof Dam, but higher concentrations than eggs from Barbers Pan. Great Cormorant eggs from Sweden and Germany (Nordén et al. 2013; Rüdel et al. 2011) had lower PFOS concentrations than Reed Cormorants eggs from Bloemhof Dam, but higher concentration than those quantified in eggs from Potchefstroom. Only eggs of Blue Herons (*Ardea herodias*) collected in 1993 in the USA near a PFAS source (Custer et al. 2010) had similar PFOS concentrations than eggs from Bloemhof Dam. PFOS concentrations at Bloemhof Dam were therefore extraordinary high considering the absence of any local source.

ΣPCB concentrations were three orders of magnitude lower in eggs from the OSRB compared with internationally reported data (Table 4), especially when comparing similar species and guilds. Grey Heron eggs from Bloemhof Dam had ΣPCB concentrations two orders of magnitude lower than concentrations quantified in France (de Cruz et al. 1997), and one order of magnitude lower than eggs from Romania (Aurigi et al. 2000). A broad observation suggests a worldwide decline in PCB concentrations. Using the Grey Heron as example, the PCB concentrations from 1970s to late 1990s were up to two orders of magnitude higher compared to post 2000 studies (Table 4). This decline was also observed in double-crested Cormorants eggs in Canada, where there was an order of magnitude decline in PCDD/F concentrations from 1987 to 1997. Long-term monitoring of POPs in eggs can aid in assessing patterns and distribution profiles (Harris et al. 2003). We could not find a study that reported ΣPCB concentrations of similar species in African studies. However, free-range chicken eggs from Tanzania had even lower ΣPCB concentrations than in the current study (Polder et al. 2016).

ΣPCDD/F concentrations reported in eggs from other regions were generally lower or of the same order of magnitude than concentrations quantified in the current study (Table 4). In addition, a number of eggs had one order of magnitude higher ΣPCDD/F concentrations (Black-headed Heron: 9 ng/g wm) than eggs from other studies (Tables 3 and 4). Eggs of double-crested Cormorants and Blue Herons from Canada (Elliott et al. 2001; Harris et al. 2003) measured the highest PCDD/F concentrations (1 ng/g wm) outside South Africa. We did not anticipate that bird eggs from South Africa would have the highest measured PCDD/F concentrations. Furthermore, all PCDD/F concentrations reported from international studies pre-date (1973–2000) the current data (2009). More recently reported PCDD/F concentrations in yellow-legged gull eggs (*Larus michahellis*) from in Spain (0.01 ng/g wm; Morales et al. 2016) and chicken eggs from Canada were also lower (Rawn et al. 2012). The current study is the first and only to report PCDD/F concentrations in wild bird eggs from South Africa, and to the best of our knowledge, also in Africa. TEQ values will be discussed in Sect. "Possible adverse consequences".

### Hotspot Identification

The ΣPCB and ΣOCP concentrations in all species were lower than those previously reported from nearby locations except for Cattle Egret eggs which were of the same order of magnitude (Bouwman et al. 2008 and 2021; Polder et al. 2008). However, African Sacred Ibis from Eldorado Park had lower ΣPCB, but higher ΣOCP concentration than those reported from Gauteng and Northern Free State (Bouwman et al. 2021) (Table 4). In addition, the African Darter concentrations from those studies were an order of magnitude higher than the African Sacred Ibis concentrations. The Gauteng concentrations reported, had among others, eggs from a colony near Eldorado Park. This may at first suggest a decrease in ΣPCB and increase in ΣOCP concentrations. However, eggs from some localities were pooled confounding interpretation. The ΣOCP concentrations were four orders of magnitude higher in Grey Heron eggs, and one order of magnitude higher in Cattle Egret eggs from areas of the country where DDT is still used (Bouwman et al. 2013) compared with the current study’s locations where DDT has been banned since 1976 (Bouwman 2004).

∑PBDE concentrations in the current study were of the same order of magnitude or lower than those reported by other authors (Table 4). African Sacred Ibis eggs collected in Eldorado Park had slightly lower ΣPBDE concentrations than those reported from nearby Soweto (Quinn et al. 2020) and Johannesburg (Bouwman et al. 2021). African Darter eggs from Gauteng (Bouwman et al. 2021; Quinn et al. 2020) had an order of magnitude higher ΣPBDE concentrations than those reported from Bloemhof Dam and Barbers Pan of the current study.

Elevated PFOS concentrations were quantified at high concentrations in bird eggs, especially at Bloemhof Dam (Table 3). To our knowledge, there is no production of PFAS in South Africa, much less in the vicinity of Bloemhof Dam, which has no industries close by. This location appears to be a hotspot for PFOS, since PFOS was found to be the dominant PFAS quantified in adult Odonata from there (median: 16 ng/g wm) (Lesch et al. 2017). Bloemhof Dam is a large impoundment, and it is possible that PFAS residues from upriver sources, accumulate at this location. Furthermore, recent published research suggests that PFAS accumulate at the air–water interface in the subsurface layer of freshwater (Brusseau 2018; Stults et al. 2023). In addition to PFOS, high concentrations of mercury (Hg) were also quantified in Great White Egrets eggs from Bloemhof Dam (van der Schyff et al. 2016). No other studies from South Africa reported on PFOS or PCDD/F concentrations in bird eggs of similar species. Compared with international reports, the high PCDD/F concentrations of the current study points towards a PCDD/F hotspot. Eggs from Barbers Pan had the highest PCDD/F concentration (9 ng/g wm; Table 3), this is concerning since this location is a Ramsar site. The four highest measured PCDD/F concentrations (BBH: 9 ng/g wm, ASI: 7 ng/g wm, BHH: 6 ng/g wm, and AD: 5 ng/g wm; Table 3) were in eggs from all four sites and from three different feeding and habitat guilds making it difficult interpret. It is concerning that concentrations quantified in eggs from all four sites were higher than internationally reported concentrations (Table 4), especially since Barbers Pan is a Ramsar site.

The data reported here represent the most current published insight into the pollution load of heron, ibis, egret, darter, and cormorants that breed in the OSRB. All other published reports were conducted during or prior to the current study. Chlordane and mirex were previously quantified in eggs of similar species (Bouwman et al. 2008; Polder et al. 2008). Studies conducted in the same year as the current collection also found quantifiable concentrations of chlordane and mirex in eggs from Gauteng (Bouwman et al. 2021) and Limpopo (Bouwman et al. 2013). These compounds were also quantified in Little Egret and White-breasted Cormorant eggs collected in 2013 in KwaZulu-Natal that is not in the OSRB. The lack of quantifiable concentrations of these compounds may be due to concentrations below LOQ.

Therefore, POP hotspots identified in this study were Bloemhof Dam for PFOS, and Eldorado Park (Gauteng) for most other POPs. We could not identify PCDD/F hotspots due to similar high concentrations detected at all four sites, nor could we explain these concentrations based on guilds. It would be reasonable to assume that the Vaal River catchment is a hotspot for PCDD/Fs, in general, and that more localised investigations need to be conducted. Bloemhof Dam has no associated industrial activities but is located approximately 450 km downstream of the most industrialised centre in the OSRB where African Sacred Ibis eggs were analysed. Eggs from Eldorado Park (in the industrialised centre) had factors to orders of magnitude higher concentrations of all compound classes at any other site, except for PFOS at Bloemhof Dam. The PFOS and PCDD/Fs results from Bloemhof Dam show that single-species studies cannot represent the picture of total avian exposure and risks as was also found by Bouwman et al. (2021). Also, assumptions about proximity to sources should not be assumed as the only factor when identifying hotspots.

### Possible Adverse Consequences

The low TEQ values in the eggs from Eldorado Park were unexpected, considering its proximity to industry. The high PCDD/F TEQ value from Barbers Pan was higher than expected, due to its isolation, remoteness from sources, and protection status as a nature sanctuary. However, the Black-headed Heron eggs from this location did have the highest quantifiable PCDD/F concentration (Table 3). Bloemhof Dam on the other hand had the highest PCB TEQ value, possibly a result of compounds accumulating at this point in the Vaal River. Bird embryos and foetuses are more sensitive to POPs than adults. Furthermore, exposure before organ development results in greater consequences when exposed after organ development (Caralson and Duby 1973). However, the TEQ values reported in this study were low compared to others (Harris et al. 2003; Hart et al. 1991). The TEQ values calculated for double-crested Cormorant eggs close to a pulp mill were up to three orders of magnitude higher than any TEQ value of the current study (Harris et al. 2003). The double-crested Cormorant hatchlings had elevated ethoxyresorufin-O-deethylase (EROD) activity and showed brain asymmetry. In addition, it is suggested that neurological activities in bird eggs are more effected by PCDD/F TEQs (Henshel 1998), which were lower than PCB TEQs in the current study. In Blue Heron eggs from Canada, a no-observed-adverse-effect-level (NOAEL) of 18 ngTEQ/kg wm was reported for developmental defects and reduced fledging (Hart et al. 1991), 10 ngTEQ/kg wm for intercerebral brain asymmetry (Henshel et al. 1995), and 100 ngTEQ/kg wm for gross abnormalities and oedema (Sanderson et al. 1994). A NOAEL of 200 ngTEQ/kg wm of coplanar PCBs were reported for Forster's tern eggs (*Sterna forsteri*) for reduced hatching success and fledging (Kubiak et al. 1989). These TEQ values were all well above all TEQ values from the current study, and therefore, we do not expect any adverse effects in eggs, hatchlings, or fledglings.

The number of PCB congeners measured affect the reported concentrations in bird eggs. While we investigated 18 congeners of which 12 are DL-PCBs and six NDL-PCBs, far higher ΣPCB residues were quantified in eggs that were investigated for fewer congeners. Field studies found mortality in double-crested Cormorant eggs at ΣPCB_?_ of 30 000 ng/kg wm (Tillitt et al. 1992) and developmental defects in Black-crowned Night herons at ΣPCB_?_ concentrations of 800 ng/g wm (Hoffman et al. 1986).

The probability of adverse effects on birds was investigated by comparing the concentrations quantified in the eggs to the available toxic reference values (TRVs) of POP. Unfortunately, TRVs are not available for all species. However, quantifiable concentrations can be compared to TVRs for other species, although it should be noted that these values are estimations and can differ greatly among species due to behavioural and biological differences. The highest ΣPCB concentrations quantified in any egg from the present study (African Darter: 102 ng/g wm) were much lower than the TRV that was derived by Hoffman et al. (1986) and Tilliet et al. (1992). We, therefore, do not expect adverse effects in birds as a result of PCB exposure for the regions sampled.

The HCB concentrations measured in bird eggs were low compared to other studies (Table 3). This is reassuring, since HCB is known to be very toxic to birds. The HCB concentrations measured in all eggs from the current study were far below the NOAEL of 1500 ng/g wm for herring gull embryos (*Larus argentatus*) embryos (Boersma and Ellenton 1986). DDT, specifically the metabolite *p,p’*-DDE, reduces eggshell thickness in eggs and may lead to reproductive failure and population decline (Dirksen et al. 1995; Peakall 1993). In Snowy Egrets (*Egretta thula*), it was found that DDE residues of 5000 ng/g wm in eggs caused thinner eggshells and lower hatching success (Findholt 1984). The *p,p’*-DDE residues in Little Egret eggs (19 ng/g wm) from Bloemhof Dam were well below this TRV. Additionally, DDE residues of 8000 ng/g wm were found to increase eggshell breakage in populations of Black-crowned Night Heron (*Nycticorax nycticorax*) populations (Henny et al. 1984). The *p,p’*-DDE concentrations of all species in the current study were well below that threshold. However, it should be noted that eggshell thinning can occur at lower exposure concentrations. Eggshell thinning has been observed in Cattle Egret eggs with increased *p,p’*-DDE and *p,p’*-DDT concentrations of up to 290 ng/g wm (Bouwman et al. 2013). For insecticide POPs, therefore, we do not expect adverse effects in birds for the region sampled.

Certain factors may influence the residue concentrations quantified in bird eggs such as the diet, habitat preference, age, and health of the female bird, as well as the time and the number of eggs laid in the clutch (Dennis et al. 2021; Mineau 1982). In addition, bioaccumulation of PFOS is 1.8 times greater in eggs when exposed through drinking water compared to food (Dennis et al. 2021). The concentrations of PFOS quantified in all eggs of all species from Bloemhof Dam and Eldorado Park (African Darter: 2330 ng/g wm) were two orders of magnitude above the TRV of PFOS estimated for Bobwhite quail (*Colinus virginianus*) whole egg (92.4 ng/g wm) (Dennis et al. 2021). The predicted no-effect concentration (PNEC) for PFOS in Bobwhite quail, regrading chick survival is 1700 ng/g wm (Newsted et al. 2005). African Darter eggs from Barbers Pan and Reed Cormorant eggs from Potchefstroom exceeded the TRV and PNEC for Bobwhite quail. PFOS therefore poses a risk for adverse effects in birds for all regions sampled.

The ΣPBDE concentrations from African Sacred Ibis eggs from Eldorado Park were 20 ng/g wm, well below the NOEL of 1000 ng/g wm for the Osprey (*Pandion haliaetus*; Chen et al. 2010). The Osprey is a high-trophic level species when compared with the African Sacred Ibis. It may be that higher PBDE concentrations will bioaccumulate in higher trophic level species from Eldorado Park. However, PBDE does not pose a risk for adverse effects in bird species sampled.

## Conclusions and Recommendations

Concentrations of all compounds detected in eggs were generally lower or of the same magnitude than those reported by most local and international studies, except for PCDD/F and PFOS. Organochlorine compounds and PCB concentrations were lower than previously reported, suggesting a decrease in the environment. Differences in POP concentrations in wild bird eggs were found between species, sites, habitat guilds, and feeding guilds. This was to be expected since species from the same region have different life histories combined with the different sources and chemical and physical characteristics of POPs.

Large aquatic predators had greater OCP and PFOS concentrations compared to species that prey on insects, while PCBs and PCDD/Fs were more prominent in terrestrial species. No patterns were observed for the other compound groups. It is recommended that additional species that occupy other feeding guilds, such as seedeaters and frugivorous, be included in future studies. It would also be instructive to determine trends and patterns over multiple years. DDT residues in bird eggs remain high in malaria endemic regions. However, Gauteng appears to be an OCP (*p,p’*-DDE) and PCB hotspot. It would be reasonable to assume that, given the lower *p,p’*-DDT concentrations and or lack of quantifiable concentrations in pooled eggs, that the ΣDDT quantified is of legacy use.

PFOS concentrations were observed to peak towards the west in the area of the Bloemhof Dam. The quantified concentrations are comparable to the concentrations detected near a PFAS source. It appears that Bloemhof Dam acts as a ‘retainer’ or ‘trap’ of some compounds coming from upstream or may reflect a local unknown source of PFOS. These concentrations pose a risk of adverse effects and should be monitored. It would be very informative to sample additional locations such as Upington downstream of Bloemhof Dam, especially with respect to the distribution of PFOS. PCDD/F concentrations quantified were unexpected. Due to the widespread occurrence of high PCDD/F concentrations, it is difficult to pinpoint specific hotspots. However, the high ΣPBDE concentrations of PBDE in Barbers Pan are concerning since it is a Ramsar site. Therefore, we recommend that more samples be collected and tested for PCDD/Fs. Overall, Bloemhof Dam would be a good monitoring site for all POPs, given its remoteness from large sources and high breeding density.

The OCP concentrations detected in bird eggs were below known TRVs. ΣPBDE concentrations in wild bird eggs were also low. However, the higher ΣPBDE concentrations from Eldorado Park are concerning and this site should be regarded as a PBDE hotspot. The combined concentrations of POPs may have greater consequences than individual POPs. Furthermore, since 2010, 13 POPs have been added to the Stockholm Convention on Persistent Organic Pollutants (Stockholm Convention 2016b), and others are in the process of being added. These new POPs need further investigation to determine possible threats and hotspots. PCB and PCDD/F concentrations and TEQ values in wild bird eggs were low, and no adverse effects are expected. Therefore, we conclude that single-site and single-species studies would not effectively represent risks representative of the complexity of avian diversity as environmental, behavioural, and physiological differences of species. Therefore, this study provides a data-rich baseline against which trends since 2010 can be investigated, especially in the hotspots and bird species reported here.

## Supplementary Information

Below is the link to the electronic supplementary material.

Supplementary file1 (DOCX 1245 KB)
