# Supplementary material for: Dioxins, PFOS, and 20 other Persistent Organic Pollutants in Eggs of Nine Wild Bird Species from the Vaal River, South Africa
Source: Arch Environ Contam Toxicol. 2024 Sep 19;87(3):287–310. doi: 10.1007/s00244-024-01088-4 (PMC11525409; doi:10.1007/s00244-024-01088-4)
Supplement: Supplementary file 1 — Supplementary file1 (DOCX 1245 KB) [file 244_2024_1088_MOESM1_ESM.docx]

**Dioxins, PFOS, and 20 other persistent organic pollutants in eggs of nine wild bird species from the Vaal River, South Africa**

Velesia Lesch, Rialet Pieters, Hindrik Bouwman

Research Unit: Environmental Sciences and Management, North-West University, Potchefstroom, South Africa

**Supplemental materials**

Table S1: General distribution and description, habitat preference, breeding behaviour, diet and egg description of bird species investigated during this study.

| **Species name** | **Distribution** | **Habitat and breeding** | **General Description** | **Foraging and Diet** | **Egg description** | **Egg mass** | **Mean egg dimensions** | **Depiction of species**  **Images by Dr W. Tarboton** |
| --- | --- | --- | --- | --- | --- | --- | --- | --- |
| Grey Heron (GH) *Ardea cinerea* | Europe, Africa, Asia, East indies islands | Aquatic. Usually located near water. Colonial nester in tall tree patches. | Large heron having white and black accents, a white crown with black plumes, black belly, and white thighs. | Large aquatic predator. Hunts fish in solitary and feed at all times during the day. | Oval, slightly pointed at both ends. Pale blue eggs | 61 g | 57-61 x 41-43 | 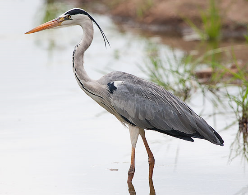 |
| African Darter (AD) *Anhinga rufa* | Africa, Madagascar, and Iraq | Aquatic. Colonial, nests in trees near water | Dark coloured bird with a thin white lateral neck stripe, pointed bill | Large aquatic predator. Dives to actively catch fish. Will also consume frogs and molluscs. | Eggs are elongated, white in colour and normally smooth. | 37g | 53 x 35 mm | 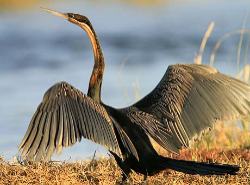 |
| Black-headed heron (BBH) *Ardea melanocephala* | Africa | Terrestrial. Breeds in tall trees or reed beds in mixed species colonies. | Medium sized bird with a dark grey, black and white huge. | Small aquatic predator. Solitary feeders, primarily insectivore, but do occasionally feed on reptiles, fish and amphibians. | Oval, pale blue eggs | 60g | 60 x 43 mm | 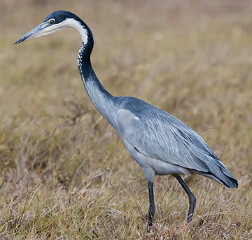 |
| Great White Egret (GWE) *Ardea alba* | Located in the Americas , eastern Europe, Africa and northern Asia | Aquatic. Breeds near water between vegetation. | Large slender white bird with a long neck, dark legs and long black plumes when breeding | Large aquatic predator. Ambushes pray by impaling its long sharp bill into prey. | Smooth, pale greenish blue. | 61 g | 56-61 x 40-43 mm | 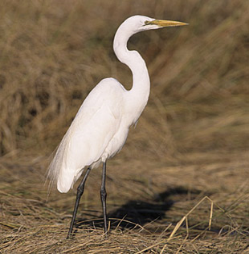 |
| Reed Cormorant (RC) *Microcarbo africanus* | Africa | Aquatic. Colonial, breeds near water in trees. Also on bare rock islands. | Small barred-backed black bird with short legs and a short dark crest above its bill. | Large aquatic predator. Hunts by propelling its feed in the water. | Eggs are a chalky blue colour | 21 g | 44 x 29 mm | 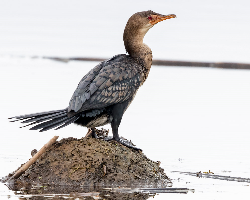 |
| African Sacred Ibis (ASI)  *Threskiornis aethiopicus* | Africa | Wetland. Colonial, nests in trees near water. | All white body with a black bald head and neck, thick curved bill and legs. | Scavenger. Feeds on many organisms and will feed on other food sources not utilize by other species. | White with red/brown spots | 62 g | 66 x 44 mm | 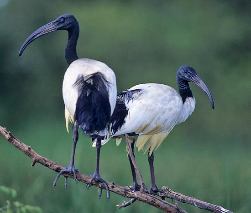 |
| Little Egret (LE) *Egretta garzetta* | Europe, Africa, Madagascar, Asia, east indies, Australia, pacific ocean island, and west indies | Aquatic. Colonial breeder that nest in trees, bushes and reed beds | Small white heron with a slender black beak, long black legs and yellow feet. | Small aquatic predator. Hunts solitarily and feed on lower aquatic invertebrates. Will occasionally hunt fish. | Oval shaped blueish green white eggs | 28 g | 47-34 mm | 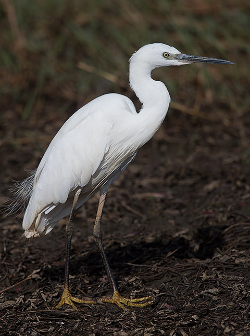 |
| Cattle Egret (CE) *Bubulcus ibis* | Worldwide distribution, found on all continents except Antarctica | Terrestrial. Colonial, breeds near water in trees and reed beds. | Medium sized bird with white feathers and a short yellow bill | Terrestrial insectivore. Feeds along large mammals or in groups usually during the day | Oval shaped blueish white eggs | 27 g | 45 x 34 mm | 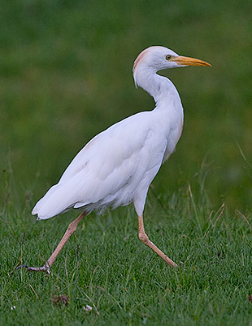 |
| Glossy Ibis (GI) *Plegadis falcinellus* | Americas, Europe, Asia, Africa and Australia | Wetland. Nests in trees and other vegetation’s near water | Medium sized ibis with reddish-brown feathers and shiny bottle-green wing feathers. Brownish bill | Small aquatic predator. Primarily feeds on insects but also consume other small organisms. | Bright blue/green eggs | 34 g | 47-58 x 33-43 mm | 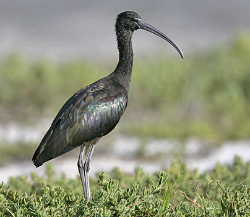 |

Table S2: Concentrations expressed as ng/kg wm of all individual congeners in pooled bird eggs samples.

Table S3: Results depicted in lipid mass
